# Decellularisation and histological characterisation of porcine peripheral nerves

**DOI:** 10.1002/bit.25964

**Published:** 2016-03-30

**Authors:** Leyla Zilic, Stacy‐Paul Wilshaw, John W. Haycock

**Affiliations:** ^1^ Faculty of Biological SciencesSchool of Biomedical SciencesUniversity of LeedsLS2 9JT United Kingdom; ^2^ Institute of Medical and Biological EngineeringSchool of Mechanical EngineeringUniversity of LeedsLS2 9JT United Kingdom; ^3^ Department of Materials Science and EngineeringKroto Research InstituteUniversity of SheffieldBroad LaneSheffieldS3 7HQ United Kingdom

**Keywords:** nerve, decelluarised, tissue engineering, Schwann cell

## Abstract

Peripheral nerve injuries affect a large proportion of the global population, often causing significant morbidity and loss of function. Current treatment strategies include the use of implantable nerve guide conduits (NGC's) to direct regenerating axons between the proximal and distal ends of the nerve gap. However, NGC's are limited in their effectiveness at promoting regeneration Current NGCs are not suitable as substrates for supporting either neuronal or Schwann cell growth, as they lack an architecture similar to that of the native extracellular matrix (ECM) of the nerve. The aim of this study was to create an acellular porcine peripheral nerve using a novel decellularisation protocol, in order to eliminate the immunogenic cellular components of the tissue, while preserving the three‐dimensional histoarchitecture and ECM components. Porcine peripheral nerve (sciatic branches were decellularised using a low concentration (0.1%; w/v) sodium dodecyl sulphate in conjunction with hypotonic buffers and protease inhibitors, and then sterilised using 0.1% (v/v) peracetic acid. Quantitative and qualitative analysis revealed a ≥95% (w/w) reduction in DNA content as well as preservation of the nerve fascicles and connective tissue. Acellular nerves were shown to have retained key ECM components such as collagen, laminin and fibronectin. Slow strain rate to failure testing demonstrated the biomechanical properties of acellular nerves to be comparable to fresh controls. In conclusion, we report the production of a biocompatible, biomechanically functional acellular scaffold, which may have use in peripheral nerve repair. Biotechnol. Bioeng. 2016;113: 2041–2053. © 2016 The Authors. *Biotechnology and Bioengineering* published by Wiley Periodicals, Inc.

## Introduction

The nervous system is one of the most highly organised biological systems, and is subdivided into the central nervous system (CNS) and peripheral nervous system (PNS). Injuries to the peripheral nerves are common and debilitating, affecting 2.8% of trauma patients (Noble et al., 1998). The microenvironment surrounding an injury site in the PNS is usually permissive to axonal regeneration. Over relatively short distances (less than 5 mm), axons can spontaneously regenerate (Rangappa et al., 2000). While the capacity of regeneration is a possibility for PNS injuries, complete functional recovery is infrequent and often misdirected in defects greater than 30 mm (Moore et al., 2009).

Current management of peripheral nerve injury includes direct end‐to‐end suturing of the damaged nerve ends or the use of an autologous nerve graft. Suturing is limited to a few millimetres for the repair of small defects or gaps. For longer nerve gaps this approach is not desirable because it has been demonstrated that any tension introduced into the nerve cable inhibits nerve regeneration (Millesi, 1982). Autologous nerve grafting remains the current gold standard surgical intervention for larger nerve defects (>30 mm). A dispensable nerve, usually the sural nerve (which has sensory function), is harvested and used to bridge the defect. Multiple segments are placed side by side, without tension to match the width of the injured nerve. Sensory nerves are used since they are deemed to be more expendable than motor nerves. Nonetheless, donor site morbidity and multiple surgeries are significant and avoidance of this necessity in treatment is desirable. Moreover it has been reported that the functional recovery using an autograft is at best 80% (Schmidt and Leach, 2003).

It has been widely demonstrated that the physical guidance of axons is a vital component of nerve repair and regeneration (Öchsner et al., 2010). Current research focuses on entubulating opposing nerve stumps of the severed nerve using a Nerve Guidance Conduit (NGC) (Kehoe et al., 2012). NGC's are manufactured from either synthetic, that is, polycapralactone (PCL) and polyclycolic acid (PGA) or natural materials such as collagen (Evans, 2001; Heath and Rutkowski, 1998; Pfister et al., 2011) and can be used for defects between 20 and 30 mm, and importantly eliminates the need for grafting (Bell and Haycock, 2012).

The Food and Drug Administration (FDA) and Conformit Europe (CE) have approved a relatively small number of NGCs (NeuraGen^®^, Neurolac^®^, and Neurotube^®^) for clinical peripheral nerve repair (Meek and Coert, 2008). In clinical trials, NGCs have been reported to have a comparable efficacy to autologous grafting in defects up to 20 mm (Meek and Coert, 2008). However, limitations of conduits include the production of acidic degradation products and high rates of degradation, which have been observed in Neurotube^®^; high rigidity reported in Neurolac^®^, which have caused needle breakages when suturing the NGC in place, and slow degradation rate in NeuraGen^®^ (up to 48 months in vivo which can lead to nerve compression (Meek and Coert, 2008). For nerve defects greater than 30 mm, autografts are still considered as the gold standard, and NGCs are not considered as effective for these gap sizes.

It has been hypothesised that the limited efficacy of NGCs in promoting nerve regeneration over longer distances is due to the lack of a suitable surface topography (Spivey et al., 2012). Peripheral nerves consist of axons held together by the ECM and basal lamina to form an atomically defined trunk and nerve fascicles. Evidence suggests that the basal lamina and ECM play important roles in both the development and repair of the PNS (Spivey et al., 2012). The ECM has an ability to influence cell morphology, phenotype and function through cell‐substrate interaction, as well as promoting cell–cell interactions. The basal lamina forms a close relationship with regenerating nerve fibres. A study by Ide et al. (1983) concluded that the basal lamina provides a pathway not only for the initial axonal elongation but also for the maintenance and maturation of regenerating axons (Ide et al., 1983).

An alternative approach is therefore attractive for creating nerve grafts which replicates the natural nerve architecture at a subcellular level in order to promote nerve regeneration over greater distances (>30 mm). One way of achieving this is through tissue decellularisation. This involves the removal of immunogenic components from the chosen tissue while retaining the 3D structure and ECM components. The rationale behind decellularisation is that ECM components are widely conserved across species and are also not adversely immunogenic.

A number of methods have been documented for the preparation of acellular nerve grafts from donor nerve tissue. gulThe most commonly utilised method involves a combination of physical, chemical, and enzymatic treatments. Commonly, physical treatments such as snap freezing is applied at the beginning of the decellularisation process to disrupt the cell membrane and lyse the cells. This is then followed by chemical and enzymatic treatments. Chemical treatments include detergents (ionic, non‐ionic, and zwitterionic); acid and alkaline treatments; hypo and hypertonic solutions and chelating agents such as EDTA. These treatments are used to solubilise both cytoplasmic proteins and nuclear cellular membranes. Additional enzymatic treatments such as nucleases are added for the removal of the cellular debris (Crapo et al., 2011; Gilbert et al., 2006). During the decellularisation process proteases are released from lysed cells, which can cause damage to the native ECM ultrastructure. Protease inhibitors such as Aprotinin are therefore added to solutions. Sterilisation methods for acellular tissue include incubation in acids or solvents, ethylene oxide exposure, gamma irradiation or electron beam irradiation (Hodde and Hiles, 2002).

AxoGen is the only company to have a clinically approved decellularised nerve graft on the market (Avance^®^ Nerve Graft), which uses utilises decellularised human donor allografts. Current data suggests the Avance^®^ Nerve Graft implants increases reinnervation and improves clinical outcome when compared to commercial available NGCs (Whitlock et al., 2009). A multicentre study using Avance^®^ Nerve Graft showed that out of all other commercially available products it was the only one that had the ability to support repair in motor, sensory and mixed nerve types, as well as having the ability to repair both short (5–14 mm), medium (15–29 mm), and long (30–50 mm) nerve gaps, making it comparable to an autograft (Brooks et al., 2012). However supply of human nerves for decellularisation and risk of product supply and disease transmission, may limit clinical potential.

The use of xenogeneic tissue offers an attractive alternative route to that of human tissue. A number of porcine organs are anatomically similar to human (e.g., the heart), but in particular the peripheral nerves, which contain an epineurium, perineurium and endoneurial microstructure, together with similar ECM organization and composition (Zilic et al., 2015). A number of advantages are therefore evident if regarding porcine nerve tissue for therapeutic purposes. We hypothesise that porcine peripheral nerves can provide a suitable level of physical guidance and regenerative support as nerve autografts. The aim of this study was therefore to develop a protocol for the decellularisation of porcine peripheral nerves. The efficacy of decellularisation was determined using histological, immunohistochemical and biochemical techniques. In addition, the biocompatibility and biomechanical properties of acellular nerves were investigated to establish potential clinical utility.

## Methods

### Procurement of Porcine Peripheral Nerves

Large White Yorkshire pigs (24–26 weeks old) were obtained from a local abattoir (J. Penny, Leeds, United Kingdom) within 24 h of slaughter. The tibial and peroneal nerves were dissected from the posterior compartment of the porcine leg, branching out from the sciatic nerve (as described in [Zilic et al., 2015]). The tibial nerve was observed to travel in the posterior section of the leg and the peroneal nerve in the lateral section. Excess fat and connective tissue was dissected from the nerve samples and tissues washed three times in phosphate buffered saline solution (PBS; Oxoid, Basingstoke, U.K.), containing 0.1% (w/v) ethylene diamine tetra acetic acid (EDTA; VWRi) to remove excess blood. Tissues were then stored at −80°C on Phosphate Buffered Saline (PBS) moistened filter paper for future use. All nerves were 150–300 mm in length and varied in diameter, with the peroneal nerve ranging from 2–3 mm and the tibial nerve ranging from 2–4 mm. [Throughout this paper the peroneal and tibial nerves will be referred to collectively as the “sciatic branches” unless otherwise stated.]

### Decellularisation

The decellularisation of porcine peripheral nerves was based on modification of a procedure described by (Wilshaw et al., 2012). The nerves were subjected to a freeze‐thaw cycle and individually processed in 250 mL of solution. Nerves were subjected to two cycles of hypotonic buffer (10 mM TRIS‐HCl; pH 8.0) and hypotonic buffer containing 0.1% (w/v) sodium dodecyl sulphate (SDS; Sigma) with agitation in the presence of Aprotinin (10 kIU/mL; Nordic Pharma) and EDTA (0.1%; w/v; VWRi). Each nerve was washed in PBS three times for 30 min with agitation. Each nerve was subsequently treated using Benzonase (1 U/mL; EMD Biosciences in buffer, 50 mM TRIS‐HCl, 1 mM MgCl_2_; Sigma; pH 7.5) for 3 h at 37°C with gentle agitation. Each nerve was then incubated in hypertonic buffer (1.5 M NaCl in 50 mM TRIS‐HCl, pH 7.6) with agitation. A sterilisation step, consisting of 0.1% (v/v) peracetic acid (Sigma, Poole, Dorset, U.K.) in PBS for 3 h at room temperature (20–25°C) with agitation was incorporated. Nerves were then washed three times in PBS at 4°C for 30 min each with agitation followed by a final 48 h wash in PBS at 4°C.

### Biological Evaluation of Peripheral Nerves

Segments (10 mm) were taken from either the proximal, distal or central regions (*n* = 3) of each nerve and fixed in 3.7% (v/v) formaldehyde prior to analysis. Acellular nerves were compared to native control nerves (*n* = 6). Samples were dehydrated in an automated tissue processer (Leica TP1020) before being embedded into paraffin wax (VWRi) to form histology blocks. Transverse sections (6 μm) of each nerve sample were cut using a rotary microtome (Leica RM 2125 RTF) and transferred onto slides. Sections were de‐waxed and dehydrated before staining by submerging sequentially in xylene (2 × 10 min), 100% ethanol (3 × 2 min), 70% (v/v) ethanol (2 min), and then water (3 min). Sections were viewed using an Olympus BX51 microscope and images captured using an Olympus XC50 digital camera (with Olympus Soft Imaging Solutions software).

### Haematoxylin and Eosin Staining

Haematoxylin & eosin (H&E; Raymond A Lamb Ltd., U.K.) staining of native and acellular nerve segments was used to evaluate tissue histoarchitecture. Samples were immersed in Harris haematoxylin (Thermo Fisher Scientific Ltd., U.K) (1 min) and rinsed under tap water for blueing (3 min). Slides were then immersed into eosin Y (VWR International) (3 min), dehydrated, cleared and mounted using DPX mountant before being viewed under Kohler illumination.

### Nuclei Staining

Acellular and native nerve slides were immersed into a 300nM DAPI working solution (20 μL DAPI [Sigma] in 200 mL PBS) and incubated for 10 min in the dark. Sections were then washed with PBS three times for 10 min each in the dark. The sections were then mounted with a glass coverslip and DPX mountant and stored in the dark until viewed. Sections were imaged using an upright epifluorescent microscope and samples observed under *λ_ex_* = 350 nm/*λ*_*em = *_460 nm. Micrograph images were captured using a digital camera (Image pro Plus v 5.1).

### Staining for Collagen and Elastin

Staining for collagen and elastin was carried out on the same slide. Samples were immersed in 5% (w/v) potassium permanganate (Thermo Fisher Scientific Ltd.) for 5 min and then rinsed with distilled water, and then submerged into 1% (w/v) oxalic acid for 2 min, rinsed with water for a further 4 min and submerged in 95% (v/v) ethanol and 70% (v/v) ethanol for 1 min, respectively. Samples were then stained for 1 h with Millers' stain (Raymond A Lamb Ltd.) and rinsed with 95% (v/v) ethanol, 70% (v/v) ethanol and distilled water respectively. Samples were subsequently stained with Weigert's iron haematoxylin (Atom Scientific, U.K.) for 10 min and rinsed with distilled water for 30 s for blueing. Samples were then stained with 0.1% (w/v) Picro‐Sirius Red (Sigma–Aldrich, Poole, Dorset, U.K.) for 1 h, rinsed with distilled water and blot dried. Sections were dehydrated, cleared and mounted using DPX mountant before being viewed under Kohler and polarised illumination.

### Staining Sulphated Glycosaminoglycans

Alcian Blue Periodic Acid Schiff's stain (ABPAS; Raymond A Lamb Ltd.) was used to localise sulphated glycosaminoglycans (GAGs). Samples were immersed into 1% (w/v) ABPAS (pH 2.5) for 1 min and rinsed with distilled water. The slides were then immersed in 0.1% (w/v) periodic acid solution (Sigma) for 5 min and rinsed three times with distilled water. The slides were then immersed in Schiff's reagent (Sigma) for 15 min and rinsed with distilled water for 5 min; cell nuclei were stained with haematoxylin (Gills Number 3 haematoxylin; Sigma) for 90 s. Samples were blued using tap water, dehydrated, cleared and mounted using DPX mountant before being viewed using an upright microscope under Kohler illumination.

### Immunolabelling for Laminin and Fibronectin

Tissue sections were permeabilised with 0.1% (v/v) Triton X‐100 (Sigma) diluted in PBS for 20 min. Samples were then incubated with 7.5% (w/v) bovine serum albumin (BSA; Sigma) diluted in PBS at room temperature for 60 min, followed by washing once with 1% (w/v) BSA in PBS. Omission of the primary antibody served as a negative control. Native porcine nerve served as a positive control; an isotype control antibody (IgG1, Dako) was used to verify antibody specificity.

#### Laminin

Nerve tissue samples were incubated with primary rabbit anti‐laminin antibody (polyclonal IgG; 0.01 mg/mL; Sigma, LN393, in 1% v/v; BSA) at 1:200 v/v dilution) at 4°C overnight, followed by washing three times with PBS for 5 min and then incubated with secondary FITC conjugated anti‐rabbit IgG (Abcam U.K., ab97050, 1:100 v/v) at room temperature in the dark for 1 h. Each section was washed three times with PBS for 5 min and counterstained with 300 nM DAPI in PBS and incubated for 20 min in the dark at room temperature. Sections were finally washed three times with PBS for 5 min and imaged using a Zeiss LSM510 META upright/inverted confocal microscopes (xenon arc lamp to excite FITC; *λ*_*ex*_= 495 nm/*λ_em_* = 515 nm). Nuclei were visualised using *λ*_*ex*_= 350 nm/*λ_em_* = 460 nm).

#### Fibronectin

Nerve samples were incubated with a primary anti‐fibronectin rabbit polyclonal antibody (Sigma; F3648; in 1% w/v; BSA at 1:400 v/v dilution) at 4°C overnight. Each sample was washed three times with PBS for 5 min each and incubated with secondary FITC‐conjugated anti‐rabbit IgG (1:100 v/v dilution) at room temperature in the dark for 1 h. Sections were subsequently washed three times with PBS for 5 min each and counterstained with 300 nM DAPI for 20 min in the dark at room temperature. Samples were then washed three times with PBS for 5 min and imaged using the confocal microscope an upright / inverted Zeiss LSM510 META confocal microscope (xenon arc lamp to excite FITC (*λ*_*ex*_= 495 nm/*λ_em_* = 515 nm). Nuclei were visualised using (*λ*_*ex*_= 350 nm/*λ_em_* = 460 nm).

### Biochemical Assays

Samples of the native (*n* = 3) and acellular porcine sciatic branches (*n* = 3) were lyophilized to a constant weight prior to biochemical assay for DNA, collagen and sulphated sugars content. Each sample consisting of 100 mg tissue were macerated with a scalpel blade and placed in a sterile 1.5 mL microcentrifuge tube. The samples were then freeze‐dried; the mass of each sample was recorded daily until a consistent weight was achieved (approximately 25 mg).

### DNA Quantification

DNA quantification was used to determine the total remaining DNA in porcine peripheral (sciatic branches) nerves following decellularisation by comparing the DNA levels in decellularised and fresh tissue. DNA extraction was carried out using the DNeasy blood and tissue kit (QIAgen 69504). Buffer ATL (360 μL) and 40 μL proteinase K (>600 mAU/mL), both from the QIAgen kit, were mixed with the macerated freeze‐dried tissue by vortexing and incubating at 56°C for 3 h or until the tissue was completely digested. Pulse vortexing (5–10 s) was performed every 30 min to disperse the sample. Each sample was vortexed for 15 s at the end of the incubation. Buffer AL (400 µL) from the QIAgen kit was added to the sample, and mixed thoroughly immediately by vortexing and 400 µL of ethanol (100%; v/v) was added, and mixed thoroughly immediately by vortexing.

Each sample was transferred to a separate DNeasy Mini spin column placed in a 2 mL collection tube, and centrifuged at 6,000*g* for 1 min. The flow‐through was discarded together with the collection tube. The DNeasy Mini spin column was placed in a fresh 2 mL collection tube, and Buffer AW1 (500 µL) was added to the column. The DNeasy membrane at the bottom of the DNeasy Mini spin column was dried by centrifuging at 15,000*g* for 3 min. The flow‐through was discarded together with the collection tube. The DNeasy Mini spin column was placed in a clean 1.5 mL microcentrifuge tube, and Buffer AE (200 µL) was added directly to the centre of the DNeasy membrane. After incubation for 1 min, the 1.5 mL microcentrifuge tube was centrifuged for 1 min at 6,000*g* to elute. The extracted DNA was in Buffer AE in the flow‐through. The DNeasy Mini spin column was discarded. The extracted DNA was subjected to quantification immediately or stored at −20°C until needed.

The concentration of extracted DNA was determined spectrophometrically using a Nanodrop spectrophotometer (Labtech). Buffer AE (2 µL) was used as a blank. Extracted DNA sample (2 µL) was loaded onto the Nanodrop, and the absorbance was determined at 260 nm. Three readings were taken for each sample, and the mean was considered as the absorbance of the sample. The DNA concentration (ng/µL) in the sample was directly displayed in the Nanodrop software. This concentration was used to calculate the DNA weight/tissue weight (µg/mg) according to:

$$DNA\;\;weight/tissue\;\;weight\;\;(\mu g/mg)\;=\;\frac{C\;\times \;200}{25\;\times \;1,000}$$

where *C* represents the DNA concentration in the Buffer AE (ng/µL). The volume of the Buffer AE containing extracted DNA was 200 µL. The total weight for each tissue sample was 25 mg.

### Hydroxyproline Assay

A hydroxyproline assay was carried out to determine the total remaining collagen remaining in porcine peripheral (sciatic branches) nerves following decellularisation. The procedure adopted was based on the method described by Woessner and subsequently modified by (Edwards and O'Brien, 1980). This assay is based on the oxidation of hydroxyproline by chloramine—T (sodium *N*‐chloro‐*p‐*toluenesulfonamide; [Sigma]) which reacts with *p‐*dimethylaminobenzaldehyde (Sigma) to form a chromophore that can be measured at 570 nm (Stegemann and Stalder, 1967). Prior to the assay the tissue samples were hydrolysed (hydrolysis of peptide bound hydroxyproline) in 5 mL of 6 M hydrochloric acid (HCL; VWRi) at 120°C for 4 h. For the assay a series of known concentrations was made up using primary and secondary standards. The primary standard was prepared by adding 25 mg trans‐4‐hydroxyproline [Sigma] in 25 mL assay buffer (13.3 g citric acid [VWRi], 3.2 mL glacial acetic acid [VWRi], 32 g sodium acetate [Thermo Fisher], 9.1 g sodium hydroxide [VWRi] and 80 mL propan‐1‐ol [VWRi] in 400 mL distilled water with pH adjusted to 6–6.5 using 6 M HCL. The secondary standard was prepared by diluting 1 mL of the primary standard in 9 mL assay buffer. Tissue samples were diluted 1:20 using assay buffer, standards were used directly in assay buffer and blank samples used 50 μL of assay buffer.

Fifty microliters of each standard and acid hydrolysed test solutions (diluted 1:20 in assay buffer so that readings were within the range of the assay) were added to a clear flat‐bottomed 96 well plate. One hundred microliters Chloramine T solution (1.41 g chloramine T [Sigma] in 100 mL distilled water) was added to each well and the plate was shaken for 5 min at 60 rpm on a shaker (Grant). 100 μL Ehrlich's reagent (7.5 g *p‐*dimethylaminobenzaldehyde; Sigma), 30 mL propan‐1‐ol (VWRi), 13.4 mL 60% (v/v) perechloric acid [BDH] and 6.6 mL distilled water) was added to each well and the plate was incubated at 60°C for 45 min.

The plate was read at 570 nm using a micro plate spectrophotometer (Multiskan spectrum; Thermo Scientific). The blank values (assay buffer) were subtracted from all standards and tissue samples and a standard curve of hydroxyproline concentration against absorbance was determined by interpolation from a trans‐4‐hydroxy‐L‐proline standard curve. The total collagen content was calculated by using a hydroxyproline to collagen ratio of 1: 7.69 (Ignat'eva et al., 2007).

### Sulphated Sugar Assay

A modified GAG assay according to (Farndale et al., 1986) was used to quantify the amount of sulphated GAGs present in porcine peripheral (sciatic branches) nerves following decellularisation. The assay is based on changes in the absorption spectrum of the cationic dye 1,9‐dimethylmethylene blue (DMB dye; Sigma) when bound to sulfated GAGs to form a complex (Farndale et al., 1986). The amount of GAGs present was determined by comparing the proteoglycan levels in the native tissue to the acellular tissue.

Protease papain was used to digest the freeze‐dried tissue samples by adding 5 mL of 50 IU/mL papain digestion solution (1,250 units of papain; [Sigma] in 25 mL of digestion buffer (5 mM L‐cysteine hydrochloride; [Sigma], 5 mM Na_2_EDTA; Thermo Fisher). Samples were incubated for 36–48 h in an oven at 60°C and diluted 1:50 in GAG assay buffer (68.5 mL of 0.1 M sodium di‐hydrogen orthophosphate (VWRi) and 31.5 mL 0.1 M di‐sodium hydrogen orthophosphate (VWRi) adjusted to pH 6.8) so that the absorbance of the unknown falls within the linear region of the standard curve.

GAG assay standards were prepared by diluting the primary standard (10 mg chondroitin sulphate B; [Sigma] in 10 mL GAG assay buffer) to known 40 μL of each standards, blank and tissue samples were added to a clear flat‐bottomed 96 well plate and 250 μL of DMB dye solution (0.008 mg 1,9 dimethylene blue; [Sigma] in 2.5 mL ethanol; [VWRi], 1 mL of formic acid; [Sigma[and 1 g of sodium formate; [VWRi]; volume made up to 500 mL using distilled water and pH adjusted to 3.0) was added to each well and placed for 2 min on a plate rocker (Grant). Sample optical densities were measured using a micro plate spectrophotometer (Multiskan spectrum; Thermo Scientific) at 525 nm. The blank values (assay buffer) were subtracted from all standards and tissue samples and a standard curve of chondroitin sulphate B concentration against absorbance was created using GraphPad Prism. The unknown values were interpolated using a linear regression of the standard curve.

### Biocompatibility of Acellular Nerve

A cytotoxicity assay was undertaken to assess whether the acellular nerves contained any residual SDS, which may prove cytotoxic to cells. Two different cell lines were used in this assay (detailed below). Cyanoacrylate glue (Sigma) and collagen type I (rat tail; Sigma C3867) were used as positive and negative controls respectively.

### Cell Culture

Human dermal fibroblasts (isolated from donor skin of abdominoplasty or breast reduction; donated form patients of the Royal Hallamshire Hospital, Sheffield, U.K; in accordance with local ethically approved NHS guidelines and under U.K. HTA Research Tissue Bank license number 12,179 regulations) and primary rat Schwann cells (isolated using a selective D‐valine method [Kaewkhaw et al., 2012]). Human dermal fibroblasts were cultured in Dulbecco's modified Eagle's medium (DMEM, Biosera) containing 10% (v/v) foetal calf serum (FCS; Biosera), 100 U/mL penicillin (Sigma), 100 μg/mL streptomycin (Sigma), 0.25 μg/mL amphotericin (Sigma), and 2 mM L‐glutamine (Sigma) at 37°C in 5% (v/v) CO_2_. Primary rat Schwann cells cells were grown in T75 cm^2^ flasks and cultured in MEM D‐Valine medium (Labtech International Ltd.) supplemented with 10% (v/v) FCS, L‐glutamine (1%; v/v), amphotericin B (0.25%; v/v), penicillin–streptomycin (1%; v/v) and forskolin (25 mM; Sigma). Cells were incubated at 37 °C in a humidified atmosphere containing 5% (v/v) CO_2._ The culture medium was changed once every 2 days.

Upon confluency both cell types were passaged by removing the culture medium and washed twice with PBS. 5 mL of 0.25% (w/v) Trypsin‐EDTA was added to flask and left to incubate for 2–3 min at 37°C in 5% (v/v) CO_2_ until cells detached from the surface of the flask_._ 5 mL of DMEM containing 10% (v/v) serum was then added to inactivate the trypsin and the cell suspension was transferred to a 50 mL centrifuge tube and centrifuged at 1,000*g* for 5 min. The supernatant was discarded and the cell pellet resuspended in 5 mL of supplemented DMEM. One milliliter of the cell suspension was then added to a new T75 flask and a further 9 mL of supplemented DMEM was added to the flask. The flask of cells was then incubated at 37°C in 5% (v/v) CO_2._

### Contact Cytotoxicity Assay

Samples of 5 mm^2^ acellular peripheral nerves were attached to the center of six‐well tissue culture plates using collagen type I. Human dermal fibroblasts and primary rat Schwann cells were seeded into each well at a density of 5 × 10^5^ cells per well and incubated at 37°C in 5% CO_2_ (v/v) for 48 h. Following incubation the culture medium discarded and the wells washed with PBS containing calcium and magnesium and fixed with 3.7% (v/v) neutral buffered formalin (Raymond A Lamb) for 10 min. Each well was stained with Giemsa solution (VWR) for 5 min. Each well was gently rinsed with tap water until running clear and left to air dry. The plates were examined under phase contrast microscopy using an inverted Olympus IX71 microscope. Any changes to cell morphology and confluency were recorded. All images were captured digitally.

### Biomechanical Properties of Porcine Peripheral Nerves

Freshly dissected peroneal (*n* = 5), tibial (*n* = 10) and acellular peroneal (*n* = 3) and tibial nerve (*n* = 4) samples were cut to 10 mm in length and their diameter measured using a calliper. Hydrated samples were clamped into a tensiometer (BOSE Electroforce test instruments, Minnesota, USA; using a 450 N load cell). A single pull to failure test was carried out at a speed of 0.1 mm/s. The first failure point or plateau was used to calculate the ultimate tensile strength (UTS), and the initial linear gradient was used to calculate the Young's modulus (YM). For all specimens the mean ultimate stress, mean ultimate strain and Young's modulus were determined from the initial length and area of the specimens.

### Data Analysis

All numerical values are shown as mean values ± 95% confidence limits. Statistical significance was assessed by the T‐method and *P*‐values of <0.05 were considered significant.

## Results and Discussion

The aim of this study was to develop a process for the decellularisation of porcine peripheral nerve, with minimal effect on the tissue properties. The major finding of the study identified a combination of low concentration SDS, hypotonic buffers and nuclease enzymes that can effectively remove cells from porcine tissue while retaining its endoneurial structure and important ECM components. The study also showed that the acellular nerve was not cytotoxic to cells and that the nerve retained its mechanical properties.

Various methods exist for preparing acellular nerve grafts from donor nerve tissue. The most commonly utilised methods for decellularisation of nerve tissue include cold preservation; (Evans et al., 1998), thermal (Ide et al., 1998; Gulati, 1988) radiation (Hiles, 1972) and detergent based chemical treatments (Hudson et al., 2004; Sondell et al., 1998).

Prolonged cold preservation of donor nerve tissue have resulted in the preservation of native Schwann cell basal laminae and nerve ECM. The drawback however was the long processing times (approximately seven weeks) as well as poor mechanical properties of the decellularised graft (Evans et al., 1998).

Thermal decellularisation subjects the nerve tissue to repeated freeze‐thaw cycles, rendering the graft non‐immunogenic by lysing cells (Gulati and Cole, 1994; Zalewski and Gulati, 1982). This process has been adapted and used successfully to produce acellular grafts, which were subsequently reseeded with Schwann cells (and/or fibroblasts). Studies have shown the grafts to effectively support axon regeneration in both central and peripheral nervous systems (Cui et al., 2003; Godinho et al., 2013; Hu et al., 2007, 2005).

There have however been reports that the freeze‐thawing process fails to remove the cell debris from the tissue, in addition to fracturing the basal laminae that surround the axons (Hudson et al., 2004). In vivo studies have demonstrated cellular remnants left behind from this process have led to cellular invasion in the form of Schwann cells and macrophages invading the basal lamina to clear away the debris. This in turn delays the regenerative process and further damages the basal lamina (Danielsen et al., 1995; Osawa et al., 1990; Pollard and Fitzpatrick, 1973).

Radiation has also been used as a decellularisation technique for nerve tissue. Studies have shown that this process does not damage the tissue morphology (Marmor, 1964). Nevertheless similar to thermal decellularisation, cellular debris still remains inside the basal laminae causing cellular invasion post implantation (Hudson et al., 2004).

Detergent processing techniques were developed to avoid the destructive effects of freeze thawing on the tissue ultrastructure, although freeze‐thawing methods are identified as producing non‐immunogenic segments (Gulati, 1988). Over a number of years detergent decellularisation processes have been optimised by using less aggressive chemical treatments with a method developed by Sondell et al. (1998) and colleagues representing the predominant method of chemical decellularisation for nerve tissue. Low concentration SDS has previously been used to decellularise various soft tissues including arteries (Wilshaw et al., 2012); liver (Uygun et al., 2010); heart valves (Korossis et al., 2002) and veins (Schaner et al., 2004).

The decellularisation procedure developed used a combination of chemical and enzymatic treatments, based on the process developed by (Wilshaw et al., 2012) and modified for porcine peripheral nerve. The first step of the process was a single freeze‐thaw cycle to open up the tissue ECM, increasing the susceptibility of subsequent decellularisation reagents. The nerves were disinfected and then exposed to a solution of 200 mM EDTA. EDTA chelates divalent metal ions (in particular Ca^2+^), which disrupts cell attachment to the ECM by interfering with the integrin secondary structure and consequent binding (McFetridge et al., 2004). This was followed by sequential incubation in hypotonic TRIS buffer to lyse the neuronal and Schwann cells, 0.1% (w/v) SDS in hypotonic buffer plus protease inhibitors (for inhibition of endogenous proteinases) to solubilise cell fragments. Benzonase, a nuclease enzyme was used to catalyse the hydrolysis of both deoxyribonucleated (DNA) and ribonucleated (RNA) chains. Disinfection of the tissue was carried out using 0.1% (v/v) peracetic acid for 3 h followed by extensive washing in PBS to remove residual SDS. Peracetic acid is a commonly used disinfection agent and also acts as a decellularisation agent (Gilbert et al., 2006). Peracetic acid is also widely used to disinfect surgical instruments due to excellent anti‐viral properties (Rutala and Weber, 2004). SDS is widely considered to be a good decellularisation agent and has been shown to effectively remove DNA remnants and cytoplasmic proteins, however at high concentrations there can be some disruption to the ECM ultrastructure as well as loss of collagen and GAGs was observed (Wilshaw et al., 2012).

Studies have shown the most successful nerve grafts, such as those developed by Sondell et al. (1998), which have demonstrated nerve regeneration are unable to match the regeneration rates observed with autografts, even with incorporation of Schwann cells (Frerichs et al., 2002). It has been hypothesised that the inadequate preservation of the ECM structure in these detergent‐treated decellularised graft is a contributing factor to this failure (Hudson et al., 2004). Studies using milder detergents have demonstrated superior preservation of native ECM and equivalent levels of decellularisation compared with previous chemical processing techniques (Hudson et al., 2004). The protocol used in this present study uses a ten‐fold lower concentration of SDS (0.1% w/v). It has been suggested by Booth et al. (2002) that the damage to the matrix reported in previous studies may have been caused indirectly by the proteases released as a result of cell lysis, and not the SDS directly (Booth et al., 2002), hence the incorporation of protease inhibitors into the protocol described in the current study.

Histological, immunohistochemical and biochemical characterisation of the acellular nerve were carried out to determine if the tissue maintained a native histioarchitecture and retained key ECM components. In this study biochemical quantification assays of the ECM components as well as the DNA content of the acellular nerve were undertaken. This combination of cellular and acellular measurements is of particular note, as there are limited reports of ECM and DNA quantification in other nerve decellularisation methods.

**Figure 1:**
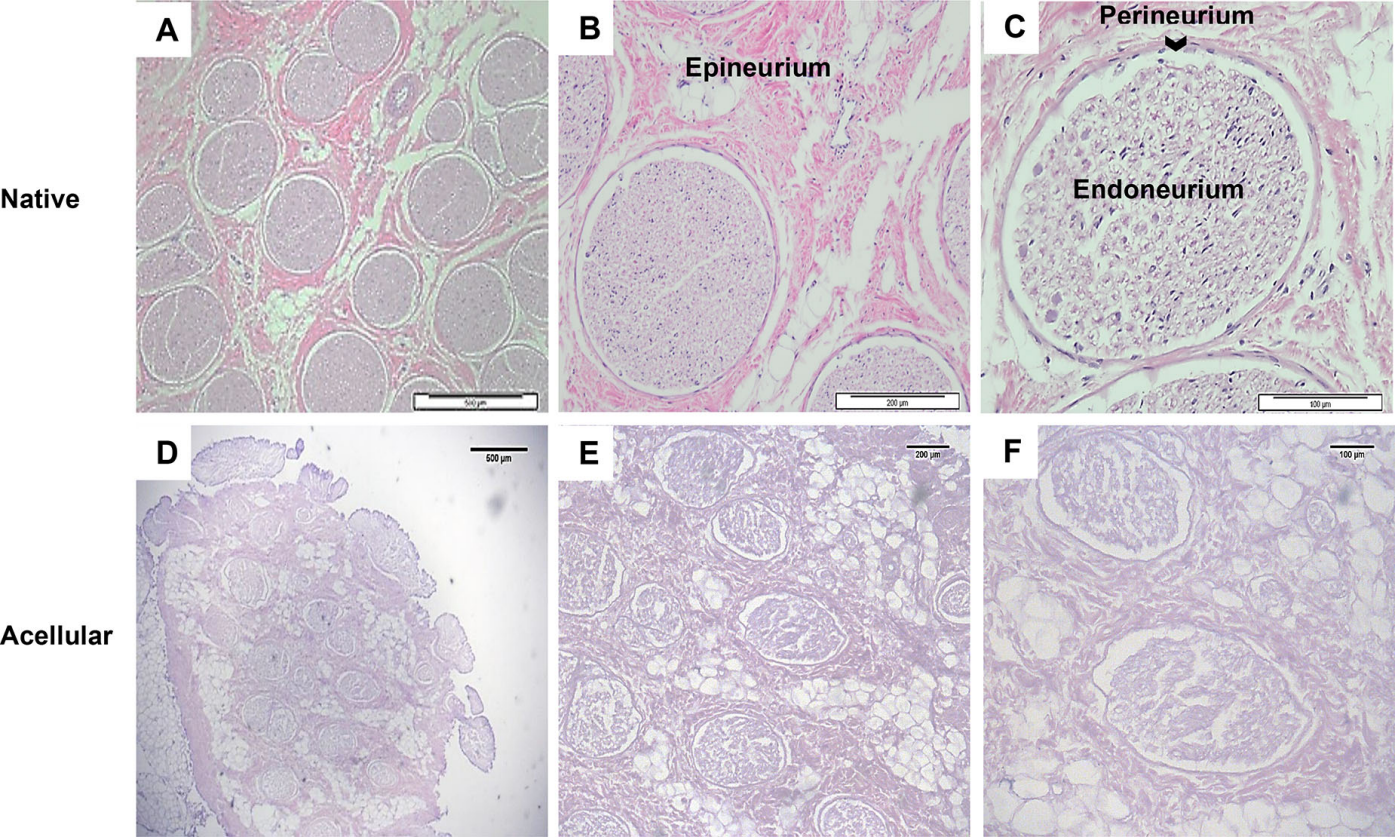
Effectiveness of the decellularisation process in the retention of tissue structure. Sections were stained with haematoxylin and eosin to identify overall histoarchitecture. Representative histological images from the central region of native (A, B and C) and acellular (D, E and F) porcine peripheral nerves. Both native and acellular nerve contains nerve fascicles along with the endoneurium, perineurium and epineurium connective tissue. Scale bars = 500 µm, 200 µm and 100 µm, respectively.

**Figure 2:**
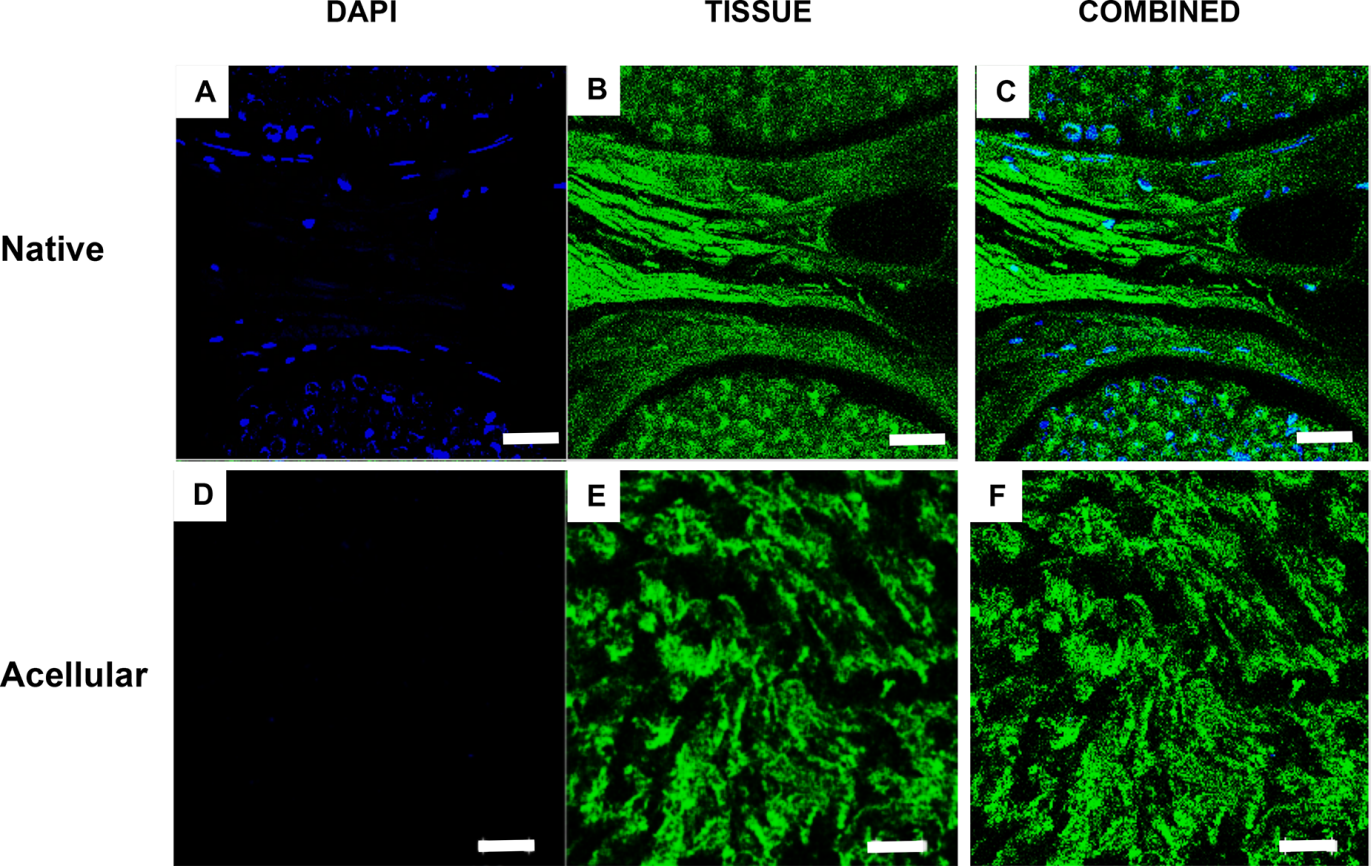
Effectiveness of the decellularisation process in the removal of cells. Sections were labelled with DAPI for identification of total cell nuclei (A & D). The surrounding green structure was the due to autofluorescence of the tissue when viewed under an upright microscope (B & E). Native nerve shows presence of cell nuclei with the endoneurium and around the perineurium, (A–C). Acellular nerve revealed a lack of cell nuclei present within its structure. Cell nuclei are shown in blue and surrounding nerve structure in green. Scale bar = 100 µm.

**Figure 3:**
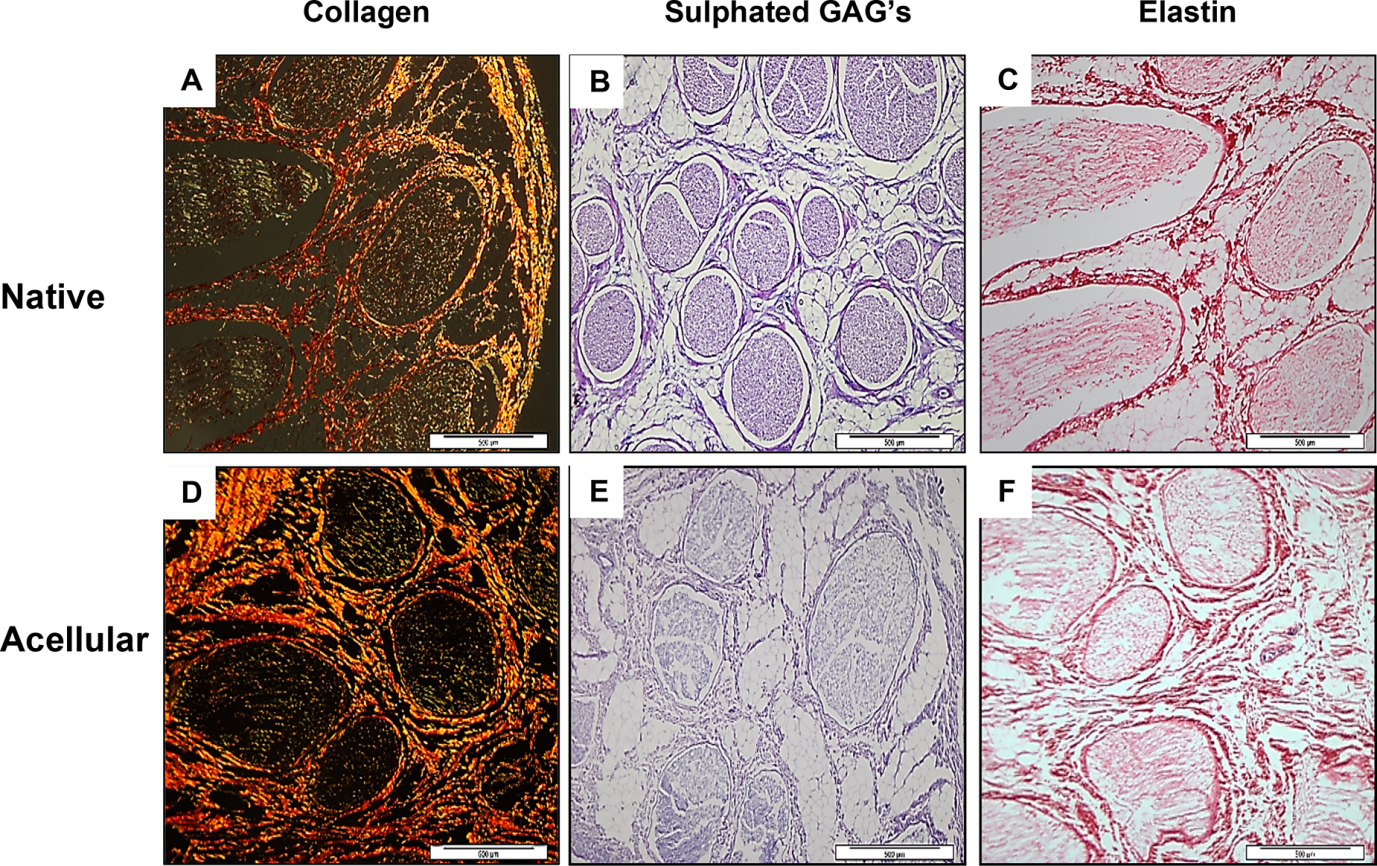
Representative histological sections from the central region of fresh and acellular porcine peripheral nerves labelled for collagen, sulphated glycosaminoglycans (GAGs) and elastin. Sections of native (A–C) and acellular sciatic nerve branches (D–F) stained using Sirius red (A & D), ABPAS Alcian Blue (B & E) or Millers stain (C & F). Staining revealed collagen retention within the endoneurium, perineurium and epineurium. GAGs were found to be retained within the endoneurium. No elastin was found to be present within native or acellular nerves. Scale bar = 500 µm.

Histological staining demonstrated retention of native nerve fascicles and connective tissues (Fig. 1) as well as collagen and sulphated GAGs (Figs. 2 and 3). Collagen is an important ECM component, constituting approximately 49% of total protein of peripheral nerve (Bunge et al., 1989). The principal role of collagen is to provide structural as well as mechanical support to the nerve. Sirius Red staining did not reveal any macroscopic or microscopic evidence of damage to collagen fibres, or significant loss in total collagen within acellular nerves. This was confirmed using a hydroxyproline assay, which demonstrated a 6.7% reduction in total collagen content when compared to the native nerve; this was not deemed significant (Table I).

**Table I:** Biochemical characterisation of fresh and acellular porcine nerves

Assay	Native nerves of dry tissue (μg/mg)	Acellular nerves of dry tissue (μg/mg)
DNA^*^	0.70 ± 0.095	0.037 ± 0.011
Collagen (hydroxyproline ×7.69)	1206 ± 195.50	1124 ± 100.70
Sulphated proteoglycans*	16.15 ± 1.30	10.81 ± 1.02

Data are expressed as mean (DNA *n* = 9; collagen *n* = 8, and proteoglycans *n* = 9) ± 95% CI. There was a significant reduction in DNA in the acellular nerve (0.037 μg/mg) when compared to the native nerve (0.70 μg/mg). There was also a significant decrease in GAG content within the acellular nerves (10.81 μg/mg) when compared with native nerves (16.15 μg/mg). There was no significant difference in the collagen content between native (1206 μg/mg) and acellular nerves (1124 μg/mg).

^*^ *P* < 0.05.

Alcian Blue staining of acellular nerves showed retention of sulphated proteoglycans (Fig. 3) however when quantified there was a 33% reduction when compared to fresh nerves (Table I). This significant reduction was thought to be due to the use of SDS. The absence of GAGs reduces the restriction on water flow and causes a reduction in the water retention of the tissue, which also facilitates the diffusion of decellularisation solutions to remove soluble cellular components (Gilbert et al., 2006). The loss of GAGs has been associated with a reduction in the ultimate tensile strength of the tissue (Partington et al., 2013). However the biomechanical testing suggested this to have a minimal effect on the tissue (Table II). While the loss in GAGs was significant, studies have indicated that chondroitin sulphate in peripheral nerves inhibits the neurite promoting activity of laminin, and therefore may have a negative role in nerve regeneration (Zuo et al., 1998). A study by Krekoski et al. (2001) treated acellular rat sciatic nerve grafts with chondroitinase ABC that inactivated CS‐GAG chains and observed an enhancement in the growth promoting properties of the acellular scaffold. Therefore the removal of GAGs may actually be beneficial in terms of its regeneration capacity.

**Table II:** Biomechanical evaluation of fresh and acellular porcine nerves

Nerve	YM (MPa)	UTS (MPa)	Strain at UTS
Native pereoneal	7.75 ± 0.56, n = 5	1.23 ± 0.06, n = 5^*^	0.23 ± 0.04, n = 5
Acellular pereoneal	8.03 ± 0.81, n = 3	1.81 ± 0.30, n = 3	0.43 ± 0.10, n = 3
Native tibial	7.43 ± 0.53, n = 10	0.87 ± 0.09, n = 10^*^	0.16 ± 0.01, n = 10^*^
Acellular tibial	8.45 ± 0.36, n = 4	2.69 ± 0.51, n = 4	0.36 ± 0.09, n = 4

YM, young's modulus; UTS, ultimate tensile stress.

Mechanical analysis showed a significant increase in UTS in both the acellular peroneal (1.81 MPa) and tibial nerve (2.69 MPa) when compared to their native nerve counterparts (native peroneal nerve; 1.23 MPa and tibial nerve; 0.87 MPa). There was a significant increase in strain at UTS for the acellular tibial nerve (0.36) when compared to its native counterpart (0.16). All data are expressed as mean (peroneal nerve *n* = 8; tibial nerve *n* = 11; acellular peroneal *n* = 3; and tibial nerve *n* = 4) ± 95% CI.

^*^ *P* < 0.05.

Millers Elastin staining indicated a lack of elastin in fresh and acellular nerves (Fig. 3). Peripheral nerves have visco‐elastic properties and are able to respond to a normal range of motion of the joints (Tassler et al., 1994). It was first suggested that the visco‐elastic properties of the nerves are due to the elastin fibres found within the connective tissue supporting elements (Sunderland, 1968). However in this study elastin was not present in the nerves. A reason for the lack of elastin detected in both the native and acellular nerve may be due to the fact that both collagen and elastin cross‐stain with the traditional histochemical techniques such as Weigert and Verhoeff‐VanGiesson, therefore making it more difficult to distinguishing the presence of elastin from collagen, however through immunolabelling, small amounts of elastin were located in the epineurium, endoneurium and perineurium, adjacent to the collagen fibres (Tassler et al., 1994). The relatively small percentage of elastin fibres found in the nerve suggests that the visco‐elastic properties of the peripheral nerve may also be due not only to elastin but to collagen as well. When viewed at a molecular level It was noted that collagen fibres were arranged in such a way to allow some degree of longitudinal stretch (Ushiki and Ide, 1990). It is thought that this specialised arrangement of layers of collagen fibres is likely to be the underlying structural component, in conjunction with fluid pressure, which provides the nerve with its viscoelasticity (Phillips et al., 2004). It was postulated by Tassler (1994) that elastin plays a role in the first phase of the stress‐strain curve (strain less than 20%) and collagen thereafter, making collagen the predominant molecule responsible for the elasticity of the nerve (Tassler et al., 1994).

**Figure 4:**
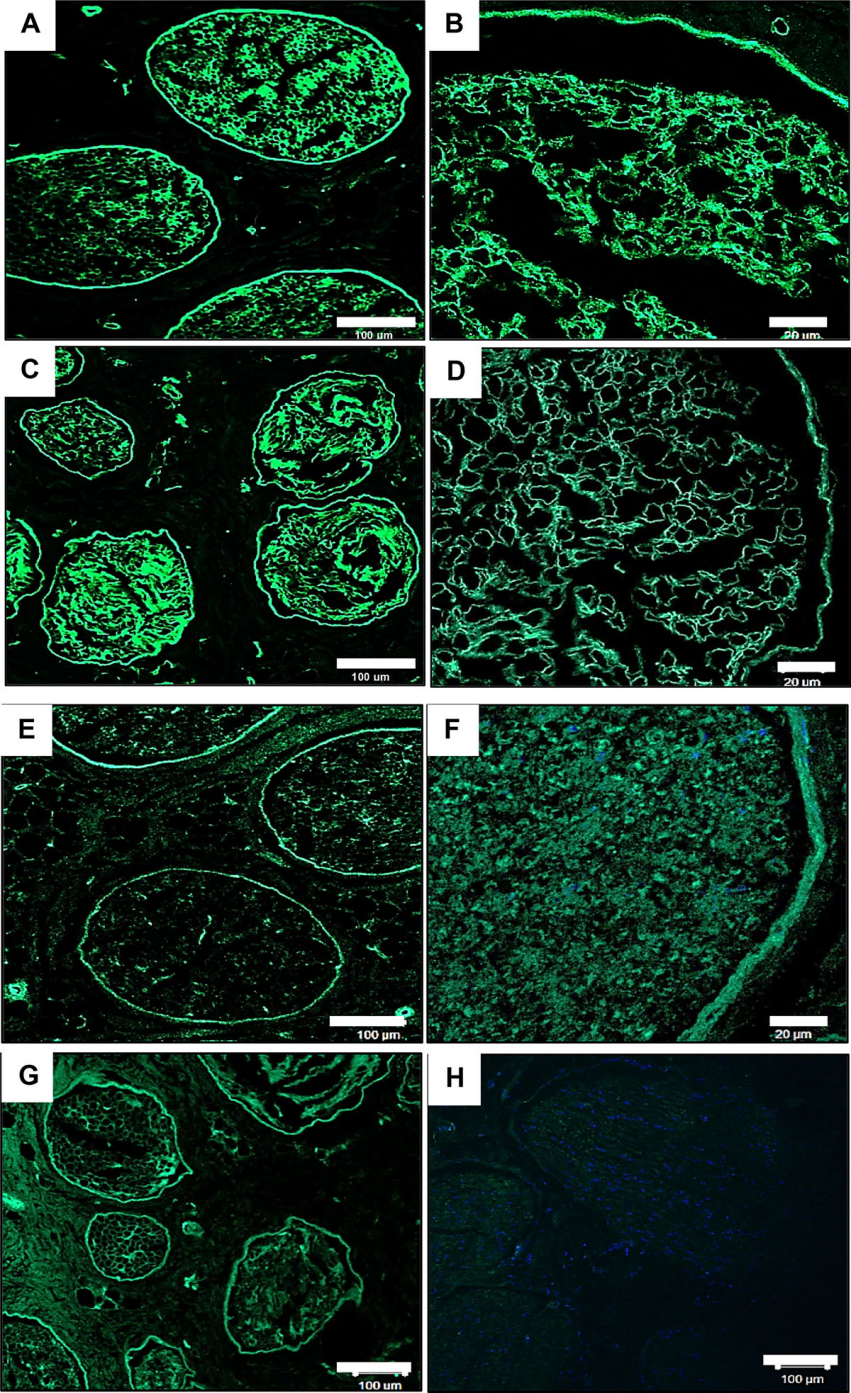
Representative histological images from the central region of fresh and acellular porcine peripheral nerves labelled using a monoclonal antibody against laminin and fibronectin. Images (A–D) show retention of laminin following decellularisation (C & D) and preservation of the basal lamina when compared to native tissue (A & B). Image G shows shows retention of fibronectin around the perineurium following decellularisation when compared to native sample (E & F). Image H is a negative control for the antibodies raised against both fibronectin and laminin (D). Scale bar = 20 µm and 100 µm.

Immunohistochemical staining revealed the retention of laminin and fibronectin within the endoneurium and around the perineurium respectively (Fig. 4). Both laminin and fibronectin are important constituents of the peripheral nerve ECM, playing essential roles in nerve regeneration (Gao et al., 2013). Laminin, which is located within the endoneurium and around the perineurium (Fig. 4A–D) has many important roles in the PNS. Along with fibronectin, laminin is a major structural element of the basement membrane as well as helping aid cell attachment (Palm and Furcht, 1983). Cellular studies have also revealed laminin enhances neuronal cell survival (Edgar et al., 1984) and guide growth cones (Dodd and Jessell, 1988). A study conducted by Lein et al. (1992) also showed that laminin had the ability to enhance axonal growth by hippocampal neurons in culture. Fibronectin, located around the perineurium and within the epineurium (Fig. 4E–G) has been shown to promote Schwann cell growth and motility, thereby enhancing regeneration of injured nerves (Ahmed et al., 2003).

A comparative study between an acellular nerve grafts produced by the Hudson detergent based method (sulfobetaine‐10; SB‐10) and Triton X‐200/sulfobetaine‐16 (SB‐16) and NeuraGen^®^ (an FDA‐approved and commercially available type I collagen NGC) saw the acellular graft outperforming the synthetic NGC (Hudson et al., 2004). The study proposed that the presence of intact basal lamina within the acellular nerve graft contributes to the enhanced regenerating capacity to support axonal growth (Whitlock et al., 2009). The method developed by Hudson et al. (2004) is currently licensed by AxoGen^®^ Inc. to produce Avance^®^.

A requisite for successful decellularisation is the removal of DNA from the tissue. Intact and fragmented DNA has the potential to elicit an inflammatory response, which can lead to rejection of the graft in vivo. Crapo et al. (2011) have proposed a decellularised graft should have less than 50 ng double stranded DNA per mg ECM dry weight as well as lack of visible nuclear material in tissue sections, which can be determined using DAPI or by H&E staining.

In the present study H&E and DAPI staining showed an absence of visible cells from tissue sections (Figs. 1 and 2) and the tissue contained 0.037 μg/mg of DNA (Table I). A study comparing the Sondell method and Hudson method used Western blotting to provide quantitative values for cellular removal of Myelin Basic Protein reported values of 0.036 ± 0.048 μg/mg and 0.054 ± 0.047 μg/mg respectively (Hudson et al., 2004). When compared to fresh nerve (1.6 ± 0.51 μg) these processes had an overall reduction in cell content of 97.75% and 96.6%, respectively. These results are comparable to the present study, in which we report a 95% (w/w) DNA reduction when compared to fresh tissue. It is important to note that the Hudson study was carried out on the rat sciatic nerve and analysed for removal of cellular protein while our study used porcine nerves and quantified total DNA content (single and double stranded DNA) in the tissue. The decellularisation process cannot eliminate total DNA from the tissue even with the most rigorous processing methods (Badylak, 2014). Most commercially available biological scaffolds contain trace amounts of remnant DNA, nonetheless the clinical efficacy of these devices have been largely positive (Table III).

**Table III:** Commercially available xenogeneic ECM scaffolds from varying source tissues and species

Source tissue	Source species	Products
Dermis	Porcine	Strattice™ (Lifecell), XenMatrix™ (Bard Davol)
Dermis	Bovine	TissueMend^®^ (Stryker), Veritas^®^ (Synovis)
Pericardium	Equine	OrtAdapt^®^ (Synovis)
Pericardium	Bovine	CopiOs^®^ (Zimmer Inc), Perimount^®^ (Edwards Lifesciences)
Small intestine	Porcine	Surgisis^®^ (Cook Biotech), Restore^®^ (DePuy Orthopaedics), FortaFlex^®^ (Integra LifeSciences)
Urinary bladder	Porcine	MatriStem^®^ (ACell)

Reproduced from (Badylak, 2014).

**Figure 5:**
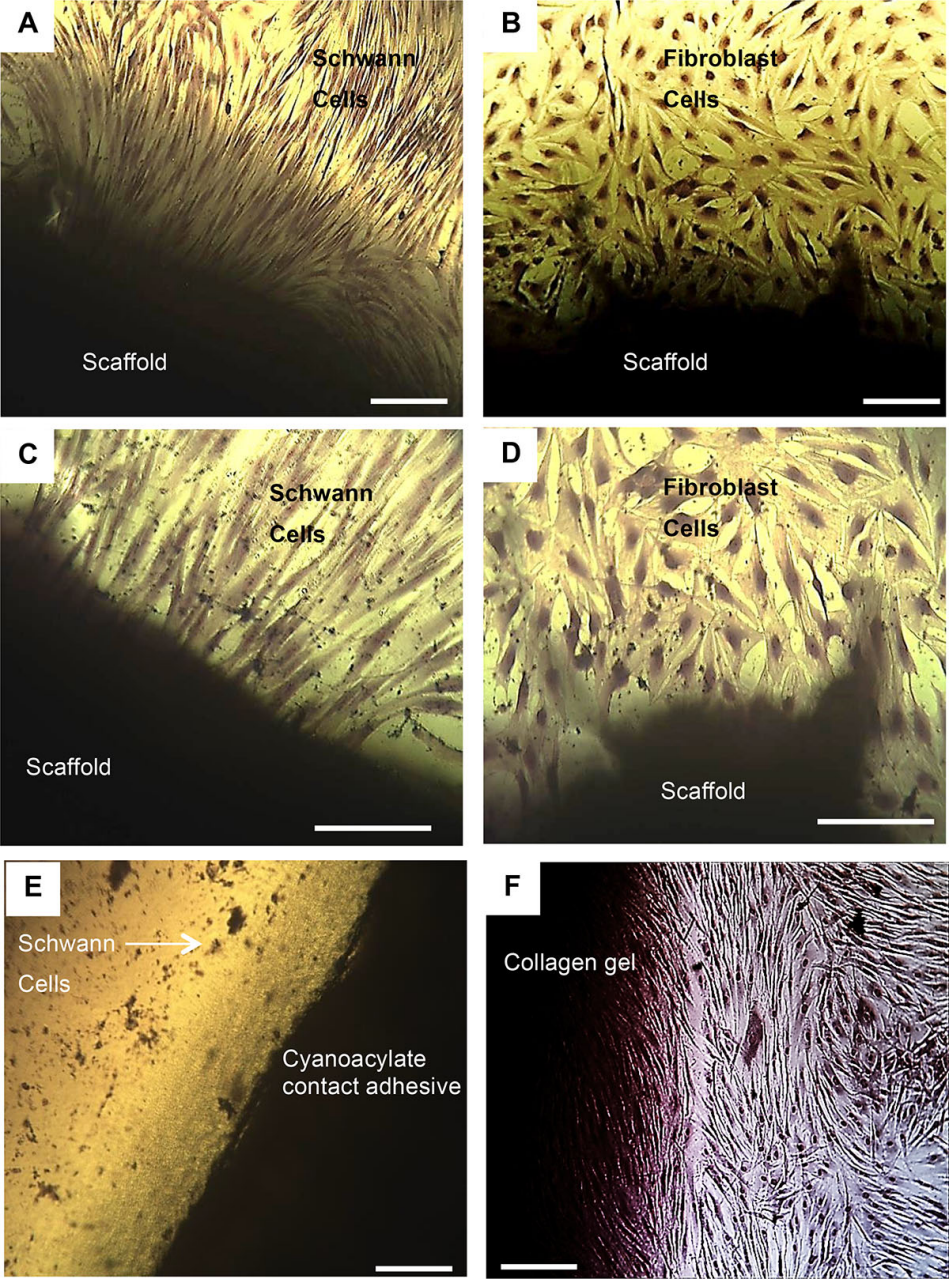
Contact cytotoxicity assays of acellular porcine nerves following 48 h culture with normal human fibroblasts and primary rat Schwann cells. The images illustrate acellular scaffolds as non‐cytotoxic to fibroblast and Schwann tested. Cells grow up to and were in physical contact with the acellular nerve for each sample (A & C). Decellularised scaffold with fibroblasts, scale bar at 100 μm and 200 μm respectively; (B & D) Decellularised scaffold with Schwann cells, scale bar = 100 μm and 200 μm respectively; (E) Cyanoacrylate contact adhesive (positive control) with the arrow indicates cells at, scale bar at 100 μm; (F) Collagen gel (negative control), scale bar = 100 μm.

SDS can sometimes be retained in decellularised tissues, which can potentially be cytotoxic to cells and therefore a contact cytotoxicity assay was performed. Both fibroblast and Schwann cells were observed microscopically and found to proliferate up to and in physical contact with the scaffold. Both cell types maintained membrane integrity, and retained structures of fibroblastic and glial phenotypes respectively. There was no sign of cell lysis, indicating that the tissue was not cytotoxic (Fig. 5).

Biomechanical testing of the acellular nerves revealed that decellularisation does have an effect on the biomechanical tensile properties, with an increase in Young's modulus, UTS and strain at UTS (Table II). There was a significant increase in the UTS for both the decellularised peroneal and tibial nerves, as well as strain for the tibial nerves (*P* < 0.05) when compared to their native counterparts. These results were found to be similar in other decellularisation studies (Abdelgaied et al., 2015; Stapleton et al., 2008; Williams et al., 2009). As previously discussed, elastin and collagen fibres contribute to the mechanical properties native nerves (Mason and Phillips, 2011; Tassler et al., 1994). During the decellularisation process the tissue becomes “looser” due to cell removal and the collagen fibre network starts to uncrimp (Williams et al., 2009). Uncrimping or relaxation of the collagen fibres has been associated with increased stiffness in soft tissues (Freed and Doehring, 2005). In addition increased fibre mobility due to the less compact nature of the acellular tissue allow collagen fibres to reorient easily towards the direction of applied strain, which would lead to increased stiffness (Williams et al., 2009).

In conclusion, porcine peripheral nerve was successfully decellularised using a low concentration SDS, hypotonic method. The acellular nerve retained its native 3D endoneurial microstructure and mechanical properties while eliminating over 95% of the cellular components. Characterisation of the ECM revealed preservation and retention of important ECM components including collagen, laminin and fibronectin. The data presented demonstrates that acellular porcine nerves may have potential clinical utility as a graft to restore motor and sensory function following injury.

We are grateful to the Doctoral Training Centre (DTC‐TERM Leeds, Sheffield and York Universities) studentship from the Engineering and Physical Sciences Research Council (EPSRC EP/F500513/1) for funding LZ. Microscope imaging was performed at the University of Sheffield (U.K.) Kroto Research Institute Confocal Imaging Facility. We are grateful to Dr Nicola Green for experimental advice and assistance with confocal microscopy.
